# Use of ropeginterferon in inducing graft versus myelofibrosis effect in post‐transplant myelofibrosis relapse

**DOI:** 10.1002/ccr3.7942

**Published:** 2023-09-21

**Authors:** Barnali Srivastava, Christine Wolschke, Nico Gagelmann, Anita Badbaran, Nicolaus Kröger

**Affiliations:** ^1^ Epworth Hospital Melbourne Australia; ^2^ Department of Stem Cell Transplantation University Medical Center Hamburg‐Eppendorf Hamburg Germany

**Keywords:** graft versus host disease, graft versus myelofibrosis, MTSS score (myelofibrosis transplant scoring system), myelofibrosis, MYSEC score (myelofibrosis secondary to PV and ET‐prognostic model), ropeginterferon

## Abstract

Here, we describe a patient with post‐transplant myelofibrosis with chronic graft‐versus‐host disease (GVHD), who showed successful molecular remission with ropeginterferon with 100% donor chimerism without any flare up of GVHD. He was initially diagnosed with polycythemia vera (PV) which progressed to myelofibrosis after 6 years. The MYSEC (Myelofibrosis Secondary to PV and ET‐Prognostic Model) and MTSS (myelofibrosis transplant scoring system) scores were 13.1 and 4, respectively, and the patient was in intermediate risk group. He underwent an allogenic stem cell transplant; however, his disease gradually progressed and was administered two donor lymphocyte infusions with minimal response. A second allogeneic transplant was performed, which led to a persistent molecular remission for more than a decade, although he developed chronic skin graft‐versus‐host disease (GVHD). The JAK2 V617F levels started to increase 10 years post‐transplant with ongoing chronic GVHD and a corresponding decrease in donor chimerism levels. He was administered ropeginterferon, which led to a decrease in JAK 2617F to undetectable levels. A graft versus myelofibrosis effect was attained with reversal to 100% donor chimerism, and he has since maintained a molecular remission with undetectable JAK 2617F levels. Chronic GVHD made him ineligible for donor lymphocyte infusions later. Thus, ropeginterferon was successful in inducing graft versus myelofibrosis effect, leading to a molecular response with no flare up of GVHD. The use of ropeginterferon needs to be further evaluated in larger cohorts of post‐transplant myelofibrosis patients.

## Graphical abstract

JAK2 level and chimerism after ropeginterferon treatment for relapsed myelofibrosis after allogeneic stem cell transplantation.

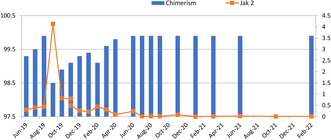

## 1 INTRODUCTION

Introducing graft versus leukemia effect (GVL) without graft‐versus‐host disease (GVHD) has always been a challenge in allogenic stem cell transplant settings. Various strategies have been tried in different clinical settings to achieve a GVL effect. Interferons have been in use for a long time in different malignancies; however, its side effects have led to decreased compliance over time. However, the availability of newer pegylated versions has made its use more convenient.

Here we discuss a case of relapsed post‐transplant secondary myelofibrosis, with chronic GVHD, successfully treated with ropeginterferon. The patient achieved a graft versus myelofibrosis effect with 100% reversal to donor cell chimerism and complete molecular remission without flare up of GVHD after using ropeginterferon.

## 2 CASE SUMMARY

48‐year‐old man initially presented to our institute in the year 2000 with a persistently raised hemoglobin (Hb) and hematocrit values for few months.

Clinical examination revealed nontender mild hepatosplenomegaly. The peripheral blood counts revealed the following: Hb 23.5 g/L; hematocrit, 0.69; white blood cell count (WBC), 10.8 × 10 *9/L; neutrophils, 79%; lymphocytes, 10%; eosinophils, 2%; basophils, 1%; monocytes, 8%; platelets, 272 × 10^9^/L. Serum LDH (lactate dehydrogenase) was normal.

Abdomen ultrasound revealed homogenous splenomegaly with a spleen size of 17 cm × 5.9 cm. The vertical dimension of the liver in the midclavicular line was 17.2 cm. There was no evidence of any lymphadenopathy.

Bone marrow evaluation revealed a markedly hypercellular marrow, with pan myeloid hyperplasia and dominating hematopoietic hyperplasia. Overall findings were consistent with a diagnosis of polycythemia vera rubra.

Cytogenetics revealed trisomy 1 and the patient was positive for JAK‐2 V617 F mutation. He was initially prescribed hydroxyurea and underwent venesections, which he well tolerated.

Six years after the initial diagnosis of PV, in 2006, he presented with generalized worsening tiredness, night sweats, and weight loss of 3–4 Kg in the last few months. Clinically, his splenomegaly had progressed, and the spleen measured 15 cm below the left costal margin, almost pressing onto the left kidney. This was accompanied by an increase in bone marrow fibrosis. Thus, his disease had progressed to post PV‐myelofibrosis.

Blood reports showed the following: Hb, 15.7 g/L; WBC, 24.0 × 10^9^/L; platelets 487 × 10^9^/L; LDH, 609 U/L.

- The MYSEC score (Myelofibrosis Secondary to PV and ET‐Prognostic Model) of 13.1 (Age 54 years = 8.1, constitutional symptoms = 1, blasts>3% = 2, non‐CARL genotype = 2). Put him in the intermediate 1 group, but with worsening splenomegaly and B symptoms.^1^ His MTSS score (Myelofibrosis Secondary to PV and ET‐Prognostic Model) was 4, and he was in the intermediate risk group.^2^

In 2006, he underwent his first HLA (human leucocyte antigen) unrelated (9/10 HLA matched) donor stem cell transplant.

The conditioning regimen used was fludarabine, busulfan, and antithymocyte globulin (ATG) according to EBMT protocol.^3^ He received 8.9 × 10*6/Kg CD34+ cells and tolerated the transplant well with no major complications or GVHD.

However, he had residual disease, which gradually progressed. A year later, in 2007, he underwent two donor lymphocyte infusions (DLI) 2 months apart with 1 × 10*7 CD3+ cells/kg; however, this produced no significant response as well as no GVHD.

In 2008, he underwent a second HLA matched unrelated alternative donor transplant after conditioning with treosulfan, fludarabine, and ATG. This time he went into persistent molecular remission and remained in remission for a long time.

However, simultaneously he developed acute gut GVHD, which initially responded to steroids. This later developed into chronic GVHD of gut and skin, and he underwent extra corporeal photopheresis (ECP) to which he partially responded.

He, however, continued to be in persistent complete remission. His JAK2 V617F levels were undetectable, and he maintained a full donor chimerism.

His disease remained in control for more than a decade, until in 2019 when his chimerism studies revealed a slight downward trend to 98.9% along with chronic moderate skin GVHD. The JAK2 V617F levels kept increasing correspondingly and increased up to 4.13 from being undetectable. Donor lymphocyte infusion was not indicated due to persistent chronic GVHD.

He was administered ropeginterferon 250 ug subcutaneously once every 2 weeks. He tolerated this well without any significant side effects for almost a year (September 2019–November 2020) and then discontinued it. He did not demonstrate any major autoimmune side effects, depression, any other significant cytopenia, or worsening of GVHD. Serial studies of both chimerism and JAK2 V617F were done. His chimerism studies gradually improved with the present study showing full chimerism. His JAK2 V617F levels became undetectable in January 2021.

**FIGURE 1:**
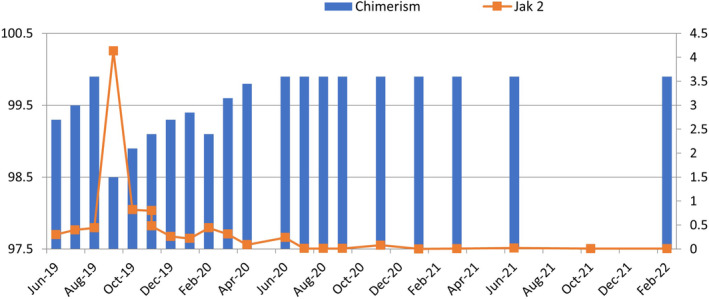
Effect of interferon on chimerism and subsequent effect on JAK2 V617F levels. Chimerism is plotted on left y axis and JAK2 V617F on the right ropeginterferon was initiated in September 2019.

Figure 1 shows a graphic representation of the effect of interferon on chimerism and the corresponding JAK2 V617F levels.

At present, the patient is not on interferon and is clinically asymptomatic with stable blood reports.

This case shows that interferon can produce a graft versus myelofibrosis effect in patients with relapsed myelofibrosis after allogeneic stem cell transplantation without worsening GVHD.

## 3 DISCUSSION

Interferons were introduced into usage a long time ago due to their innate ability to produce resistance to viral replication. Type I and 2 interferons act on human interferon alpha receptor chains and activate signaling through the JAK/STAT pathway. Interferons can also enhance natural killer and T cells, macrophage activity, and enhance expression of tumor associated and major histocompatibility complex (MHC) antigens.

Interferon has been used in myeloproliferative neoplasms for a long time. Its use in polycythaemia vera (PV) and essential thrombocytosis (ET) has led to a decrease in mutant alleles of JAK2 V617F and calreticulin (CARL) and this has led to a renewed interest in expanding its usage. Interferon can suppress clonal hematopoiesis in essential thrombocytosis and polycythaemia vera. Prior to tyrosine kinase inhibitors, interferons have been used in chronic myeloid leukemia (CML) and has induced cytogenetic remission.

The PEGINVERA study also demonstrates the effectiveness of ropeginterferon in achieving 80% complete hematological and molecular response in patients with long‐term usage.^4^ Primary myelofibrosis is a clonal myeloproliferative disorder characterized by splenomegaly, leucoerythroblastic blood picture, cytopenia, bone marrow fibrosis, and absence of Philadelphia chromosome. Secondary myelofibrosis can be a result of progression of myeloproliferative disorders, such as PV and ET.

Molecular landscape consists of JAK2 V617F, CARL, MPL, or in their absence other clonal driver mutations (ASXL1, EZH2, TET2, IDH1/IDH2, SRSF2, and SF3B1).

Till now, allogenic stem cell transplant remains the only potential curative treatment of myelofibrosis. This case described here is of post‐transplant secondary myelofibrosis, where the patient had a molecular relapse with ongoing chronic skin GVHD. He was administered ropeginterferon which was tolerated well without any flare up of his GVHD. Serial monitoring of his JAK2 V617F levels and chimerism studies revealed an excellent sustained response to treatment. He was initially diagnosed with polycythaemia vera, JAK2 V617F positive, which progressed to secondary myelofibrosis. He had minimal response to the first allogeneic stem cell transplant and donor lymphocyte infusions was administered. The disease, however, progressed, and he proceeded on to a second allogeneic transplant. He gradually developed acute followed by chronic skin and gut GVHD for which he was administered steroids and received ECP (extracorporeal photopheresis), and he maintained his remission for a long time.

A follow up after 11 years revealed a drop in chimerism and a rise in JAK2 V617F levels. However, he had ongoing chronic skin GVHD. He was administered ropeginterferon at a dose of 250 ug subcutaneously every fortnight, which continued for a year. This led to a gradual rise in chimerism and decrease in JAK2 V617F levels. His last reports (February 2022) revealed chimerism of 99.9%, and JAK2 levels of 0.006. Interestingly, his skin GVHD also responded well and did not show any flare up.

It has been observed that better responses were seen in non‐T depleted transplants highlighting the importance of donor T cells to produce a DLI effect in controlling the malignancy. In PV, interferon is known to cause a reduction in *JAK2 V617F* allele frequency; however, this effect in advanced myelofibrosis, if any, is small. The possibility of the direct tumor effect of interferon leading to the remission in our patients cannot be ruled out. However, as seen in patients with relapsed CML after allograft, the major effect of interferon postallograft is to increase HLA restricted antigen presentation, which allows better donor T‐cell mediated graft versus leukemia effect.

We have used the term graft versus myelofibrosis effect here as our patient is a case of advanced myelofibrosis.

As reported by Steegman et al, interferon has produced durable, complete cytogenic response with acceptable toxicity in relapsed patients with postallogenic transplant CML.^5^ It was also observed that when interferon is used in a molecular relapse stage it produces better durable responses compared to when it is used in an advanced hematological relapse state, and patients continued to have a prolonged cytogenetic remission of more than 5 years when followed up.

Similar studies have also shown that interferon (ropeginterferon) is quite effective in producing cytogenetic remission in relapse post‐transplant allogeneic transplant patients of CML.^6^

This emphasizes the potential of interferons in immunomodulating effect and maintaining a sustained remission in myeloproliferative neoplasms. This line of treatment can be considered in patients with persistent residual disease or relapse after allogenic stem cell transplant to increase a graft versus myelofibrosis/leukemia effect, especially if donor lymphocyte infusions are contraindicated due to GVHD.

## 4 CONCLUSION

Interferons have been in use for a long time. However, due to the side effects of the initial preparation and the access to newer molecules its use has transiently decreased. However, with the newer pegylated preparations, there has been an increase in its use in hematological malignancies. With careful considerations to the known side effects, it can be used in patients with relapsed myelofibrosis after allogeneic stem cell transplantation when DLI is practically not possible.

The use of interferon in producing graft versus myelofibrosis needs to be explored in a larger cohort of myelofibrosis patients relapsing after allogeneic stem cell transplant.

## AUTHOR CONTRIBUTIONS

**Barnali Srivastava:** Conceptualization; data curation; formal analysis; project administration; resources; visualization; writing – original draft; writing – review and editing. **Christine Wolschke:** Conceptualization; data curation; methodology; resources. **Nico Gagelmann:** Conceptualization; data curation; formal analysis; resources. **Anita Badbaran:** Conceptualization; data curation; formal analysis; resources. **Nicolaus Kröger:** Conceptualization; data curation; formal analysis; project administration; resources; software; supervision; visualization.

## CONFLICT OF INTEREST STATEMENT

The authors declare that they have no conflict of interest.

## CONSENT

Written informed consent was obtained from the patient to publish this report in accordance with the journal's patient consent policy.

## NOVELTY STATEMENT

The new aspect shown in this study is the efficient use of ropeginterferon in inducing a 100% chimerism in relapsed post‐transplant myelofibrosis. The central finding of the study is the induction of graft versus myelofibrosis effect with ropeginterferon and attaining a successful molecular remission in relapsed post‐transplant myelofibrosis cases ineligible for donor lymphocyte infusions. The specific clinical relevance of the study is considering the use of ropeginterferon to produce molecular remissions and induce 100% chimerism in patients with relapsed post‐transplant myelofibrosis.

## Data Availability

The data that support the findings of this study are available from the corresponding author upon reasonable request.
